# Development of CD8^+^ T cells expressing two distinct receptors specific for MTB and HIV-1 peptides

**DOI:** 10.1111/jcmm.12053

**Published:** 2013-04-04

**Authors:** Pei-Pei Hao, Xiao-Bing Zhang, Wei Luo, Chao-Ying Zhou, Qian Wen, Zhi Yang, Su-Dong Liu, Zhen-Min Jiang, Ming-Qian Zhou, Qi Jin, Li Ma

**Affiliations:** a Institute of Molecular Immunology, School of Biotechnology, Southern Medical UniversityGuangzhou, China; b Institute of Pathogen Biology, Chinese Academy of Medical Sciences & Peking Union Medical CollegeBeijing, China

**Keywords:** *Mycobacterium tuberculosis*, human immunodeficiency virus, co-infection, TCR gene modification, CD8^+^ T cell

## Abstract

The immune response in individuals co-infected with *Mycobacterium tuberculosis* (MTB) and the human immunodeficiency virus (MTB/HIV) gradually deteriorates, particularly in the cellular compartment. Adoptive transfer of functional effector T cells can confer protective immunity to immunodeficient MTB/HIV co-infected recipients. However, few such effector T cells exist *in vivo*, and their isolation and amplification to sufficient numbers is difficult. Therefore, enhancing immune responses against both pathogens is critical for treating MTB/HIV co-infected patients. One approach is adoptive transfer of T cell receptor (TCR) gene-modified T cells for the treatment of MTB/HIV co-infections because lymphocyte numbers and their functional avidity is significantly increased by TCR gene transfer. To generate bispecific CD8^+^ T cells, MTB Ag85B_199–207_ peptide-specific TCRs (MTB/TCR) and HIV-1 Env_120–128_ peptide-specific TCRs (HIV/TCR) were isolated and introduced into CD8^+^ T cells simultaneously using a retroviral vector. To avoid mispairing among exogenous and endogenous TCRs, and to improve the function and stability of the introduced TCRs, several strategies were employed, including introducing mutations in the MTB/TCR constant (C) regions, substituting part of the HIV/TCR C regions with CD3ζ, and linking gene segments with three different 2A peptides. Results presented in this report suggest that the engineered T cells possessed peptide-specific specificity resulting in cytokine production and cytotoxic activity. This is the first report describing the generation of engineered T cells specific for two different pathogens and provides new insights into TCR gene therapy for the treatment of immunocompromised MTB/HIV co-infected patients.

## Introduction

*Mycobacterium tuberculosis*/human immunodeficiency virus (MTB/HIV) co-infections are a challenge to the prevention and control of tuberculosis and AIDS. HIV infection represents the most significant risk factor for acquiring tuberculosis (TB) infection and TB is the leading cause of death among people living with HIV. It has been reported by the World Health Organization (WHO) that 1/8 of the 8.8 million newly infected TB patients in 2010 were co-infected with HIV (World Health Organization HIV/TB Facts 2011. http://www.who.int/hiv/topics/tb/en/). About 1/3 of MTB/HIV-infected patients deteriorate rapidly and will die in a short time. Treatment of both infections requires long courses of concomitant anti-TB and anti-retroviral drug therapies making adherence to drug treatment regimens challenging and contribute to the development of drug-resistant HIV and MTB strains 1, 2. The increasing emergence of multi-drug and extensively drug-resistant MTB and HIV strains requires design of new therapeutic options designed to enhance immune responses against both pathogens in co-infected patients.

Because MTB and HIV are intracellular pathogens humoural responses are largely ineffective compared to cellular immune responses that represent the primary protective mechanisms against infection. However, in MTB/HIV co-infected patients cellular immunity (critical to the control of MTB infection) is significantly impaired. Failure to control MTB and HIV infections is because of the rapid depletion of activated CD4^+^ T helper 1 (Th1) effector cells and impaired maturation and activity of CD8^+^ cytotoxic T lymphocyte cells (CTLs) associated with significant reductions in interferon-gamma (IFN-γ) and tumour necrosis factor-α (TNF-α) production 3–5. These impairments to CTL activity represent a major immune evasion mechanism associated with intracellular pathogens designed to avoid killing of infected host cells 6. Further complicating the development of treatment strategies is the ease by which drug resistance develops in both MTB and HIV-1 7–13. In addition, HIV-1 can also escape immune surveillance by introducing escape mutations within CTL-specific epitopes 14–16. Furthermore, these pathogens elevate each other's virulence in co-infected individuals resulting in accelerated deterioration of immunological function and accelerated mortality rates 17. It would therefore be advantageous to direct immune responses to epitopes specific to both pathogens. If one epitope is lost as a result of mutation(s), bispecific CTLs could still respond to other epitopes. Therefore, eliciting immune responses specific to both pathogens could help control MTB and HIV-1 co-infections.

It has been demonstrated that adoptive effector T cell transfer to immunocompromised or immunodeficient patients conferred protective immunity and enhanced the capacity of effector T cells to specifically recognize and kill targets *in vivo*, resulting in an improved immune status in recipients 18. This strategy contributes both to MTB clearance 19, 20 and enhanced HIV-1-specific immune responses 21. However, this approach has never been used as a therapy option in the treatment of MTB/HIV co-infected patients possibly because of the few effector T cells recoverable from these patients and challenges associated with the amplification of T cells to sufficient numbers *in vitro* for use in adoptive transfer protocols.

The T cell receptor (TCR) expressed on the surface of T cells is critical for antigen recognition and elicitation of effector functions. TCR gene transfer is a powerful tool used in the rapid generation of a large number of effector T cells with high functional avidity. Recently, TCR gene-modified T cells have been developed for the adoptive transfer treatment of leukaemia 22, metastatic melanoma 23 and cytomegalovirus 24 and Epstein-Barr virus infections 25. These approaches resulted in positive results suggesting that this approach could be developed into a promising therapeutic strategy. Our previous work demonstrated for the first time that engineered T cells with specific TCR gene-modified CD4^+^ and CD8^+^ T cells targeting the intracellular bacterial MTB 38 kDa antigen displayed enhanced functional avidity 26. Genetically engineered CD8^+^ T cells expressing TCRs specific for the HIV-1 gag epitope have been reported both *in vitro* and *in vivo* 27. These genetically engineered T cells expressing two additional receptors (TETARs) specific for two HIV-1 epitopes were also generated, and the cytokine secretion profile and cytotoxic epitope-specific functions described 28. However, modification of T cells with two additional TCRs targeting different pathogen antigens has never been reported.

During the process of TCR gene transfer, mispairing of endogenous and exogenous TCR α and β chain genes can significantly affect the expression and functional activity of the introduced TCRs. This can be prevented by using three strategies: (1) Mutation of the TCR α and β chain C regions; (2) Substituting a part of the TCR α and β chain C regions with the CD3ζ gene sequence and (3) Using different 2A sequences as gene linkers to obtain equal expression levels of TCR α and β gene fragments. It was reported that replacement of nine critical amino acids in the TCR α and β chain C regions with their murine counterparts enabled preferential pairing of the transferred TCR genes resulting in more stable binding with CD3 which resulted in enhanced expression and function of the transferred TCRs 29. In addition, substituting the TCR α and β chain C regions (downstream of the extracellular cysteine) with a complete CD3ζ gene can ensure correct TCRαβ:CD3ζ chain pairings resulting in potent intracellular signalling by activating nuclear factor of activated T cells for regulating T-cell development and function 30. To ensure stable, equal expression of multiple proteins from a single open reading frame (ORF), 2A peptides derived from picornavirus families are commonly used. 2A peptides like P2A, E3A, T2A and F2A have been derived from porcine teschovirus-1 (PTV1), equine rhinitis A virus (ERAV), Thosea asigna virus (TAV), and foot-and-mouth disease virus (FMDV) respectively. Use of different 2A peptide sequences is important to minimize the risk of homologous recombination when linking more than two genes in a retroviral vector 31, 32. The function of 2A peptides depends on the highly conserved carboxyl-terminal GDVE(S/E)NPGP sequence that mediates ‘ribosomal skipping’ between the glycine (G) and proline (P) 33. By linking TCR chains with 2A peptides expression of up to four proteins in one ORF can be achieved 31, 34, 35.

In this study, we isolated TCRs specific to the MTB Ag85B_199–207_ and HIV-1 Env_120–128_ peptides respectively. Both TCR genes were cloned into a retroviral vector and transduced into CD8^+^ T cells to generate T cells (referred to as TETARs) simultaneously expressing two additional receptors specific for two epitopes from different pathogens. Our results showed that the TETARs specifically recognized the MTB and HIV-1 epitopes and exerted anti-TB and anti-HIV-1 activities. This report describes a strategy for establishing a new type of adoptive immunotherapy based on the generation of bispecific TCR gene-modified T cells that can be used in the treatment of immunocompromised MTB/HIV co-infected patients.

## Materials and methods

### Isolation and culture of peptide-stimulated T cells

The protocol was approved by the ethics committee of the Southern Medical University. Blood samples were obtained from a HLA-A*0201 healthy volunteer with informed consent. Peripheral blood mononuclear cells (PBMCs) were isolated from whole blood by Ficoll-Hypaque (Shanghai Second Chemistry Factory, Shanghai, China) gradient centrifugation. PBMCs were seeded in 6-well culture plates (Nunc, Roskilde, Demark) in RPMI-1640 medium (Hyclone Ltd, Logan, UT, USA) supplemented with 10% foetal bovine serum (FBS; Hyclone) and 100 u/ml interleukin-2 (IL-2) (PeproTech, RockyHill, NJ, USA). At the same time, PBMCs were stimulated with HLA-A*0201-restricted peptides MTB Ag85B_199–207_ (KLVANNTRL) or HIV-1 Env_120–128_ (KLTPLCVTL) at a final concentration of 50 ng/ml on day 0, 5, 10 respectively. Both peptides were synthesized by Beijing AuGCT DNA-SYN Biotechnology Co., Ltd (Beijing, China) with a purity of 98%. After three cycles of stimulation, PBMCs were collected for CD8^+^ T cell sorting.

### Sorting of CD8^+^ T cells using magnetic beads

The Ag85B_199–207_- and Env_120–128_-stimulated CD8^+^ T cells were sorted using CD8-coated immunomagnetic beads (Miltenyi Biotec, Bergisch Gladbach, Germany) following the manufacturer's instructions. The isolated CD8^+^ T cells were stained with fluorescein isothiocyanate-conjugated CD8 mAb and detected by FACSCalibur flow cytometer using CELLQuest software (BD Biosciences, San Jose, CA, USA), the purity of these cells were >95% (data not shown).

### Screening of peptide-specific TCRs by TCR-CDR3 spectra type analysis

RNA was extracted from Ag85B_199–207_- and Env_120–128_-stimulated CD8^+^ T cells and reversely transcribed into cDNA using RevertAid™ First Strand cDNA Synthesis Kit (Fermentas, MBI, ON, Canada). The complementarity determining region 3 (CDR3) of 24 Vβ (β chain variable gene) and 32 Vα (α chain variable gene) gene families were amplified as previously described 26. Antigen-specific TCRs were identified by CDR3 spectratype analysis.

### Construction of the retroviral vectors

The retroviral vector used in this study was pMX-internal ribosomal entry site (IRES)-green fluorescent protein (GFP) retroviral vector (kindly provided by Han H, Fourth Military Medical University, Xi'an, China). An insert containing the MTB Ag85B_199–207_ peptide-specific TCRs (MTB/TCR) α13- and β16-chains and the HIV-1 Env_120–128_ peptide-specific TCRs (HIV/TCR) α11- and β18-chains was created using overlapping PCR. Nine amino acids (AA) in the C regions were replaced by their murine counterparts.

The initial PCR was performed for the MTB/TCR β16-chain using primers containing a 5-AA mutation. β primers included the wild-type β forward primer P1 with the reverse primer P2 containing the 2-AA mutation near to the amino-terminal of C region, the forward primer P3 containing the same 2-AA mutation with the reverse primer P4 containing the 3-AA mutation near to the carboxyl-terminal of C region, the forward primer P5 containing the same 3-AA mutation with the wild-type β reverse primer P6 (containing 5′-end of P2A). A second PCR used the wild-type β forward primer P1 with the reverse primer P4, and a third PCR using the wild-type β forward primer P1 with the wild-type β reverse primer P6 completed the β-chain. Similarly, the MTB/TCR α13-chain was created using the wild-type α forward primer P7 (containing 3′-end of P2A) with the reverse primer P8 containing the 4-AA mutation, the forward primer P9 containing the same 4-AA mutation with the wild-type α reverse primer P10 (containing 5′-end of T2A). A second PCR used the wild-type α forward primer P7 with the wild-type α reverse primer P10 completed the α-chain. The full-length MTB/TCR was formed in a third PCR using the β16 and α13 products with the wild-type β forward primer P1 and the α reverse primer P10.

To generate HIV/TCR β18-chain, the wide-type β forward primer P11 (containing 3′-end of T2A) with the reverse primer P12 (containing the linker sequence ggggat and 5′-end of CD3ζ) and the forward primer P13 (also containing ggggat and 5′-end of CD3ζ) with the wide-type β reverse primer P14 (containing 5′-end of F2A) were used. The primer P11 and P14 completed the β-chain. The HIV/TCR α11-chain was created in a similar fashion using the wide-type α forward primer P15 (containing 3′-end of F2A) with the reverse primer P16 and the forward primer P17 with the wide-type α reverse primer P18. The primer P15 and P18 completed the α-chain.

The MTB/TCR β16/α13 PCR products were digested with Xho I (Takara, Shiga, Japan) and Aat II in the T2A, the HIV/TCR β18-chain PCR products were digested with Aat II and Age I in the F2A, the HIV/TCR α11-chain PCR products were digested with AgeI and Not I, and the pMX-IRES-GFP retroviral vector was digested with XhoI and Not I. The HIV/TCR β18-chain and α11-chain were firstly linked by T4 DNA ligase (Takara) to form the HIV/TCR. Then the MTB/TCR β16/α13, the HIV/TCR β18/α11 and the digested vector were linked to form the construct pMX-β16-p2A-α13-T2A-β18-F2A-α11-IRES-GFP. As the controls, the constructs pMX-β16-p2A-α13-IRES-GFP and pMX-β18-F2A-α11-IRES-GFP vectors were also generated using the above primers, except that the primer P10 was substituted by P10′ (containing the restriction site of Not I but no 5′-end of T2A), and the primer P11 was substituted by P11′ (containing the restriction site of Xho I but no 3′-end of T2A). To promote the recognition of the initiator codon by ribosomes, a conventional Kozak sequence located in the 5′ untranslated messenger ribonucleic acid region was added into the primers P1 and P11′. A GSG linker ensuring complete ‘cleavage’ between the upstream cistron and the 2A peptide was added into the primers P6, P10 and P14. All of the above-mentioned primers (P1–P18) and the primers used to generate 2A-linkers are summarized in Table 1.

**Table 1:** Primers used for amplification of mutant TCRs and 2A-linked reconstitution sequences

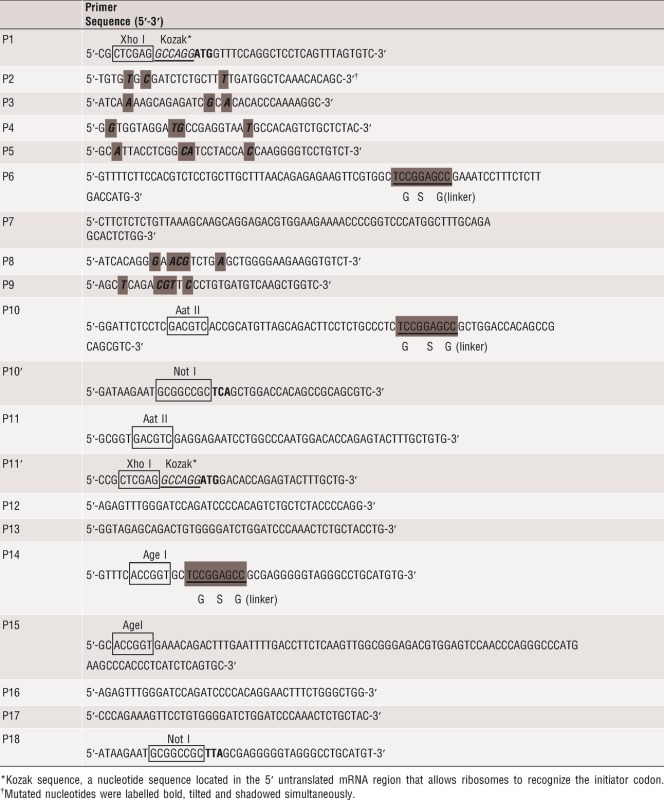

### Packaging and titre of recombinant retrovirus

Retroviral vectors containing TCR genes and VSV-G envelope protein vectors were cotransduced into GP2-293 packing cells 36 using Lipofectamine 2000 (Invitrogen, Carlsbad, CA, USA) according to manufacturer's instructions. Viral supernatants were harvested 48–72 hrs later and concentrated by ultracentrifugation at 50,000 × g for 90 min. at 4°C. The recombinant retroviral particles were resuspended in fresh serum-free medium at 0.5–1% of the original culture supernatant volume and stored at −70°C.

A quantity of 5 µl of the concentrated virus suspension containing 8 μg/ml polybrene (a cationic polymer used to increase the adhesion of virus to cells) (Sigma-Aldrich, St. Louis, MO, USA) was then applied to NIH3T3 cells, which were plated at 1×10^6^ cells/well in 6-well culture plates the day before infection. The culture supernatants were replaced with fresh culture medium 24 hrs later. Cells were harvested 3 days after infection and viral titres were measured by flow cytometry (BD Biosciences) and calculated as follows: GFP positive rate×10^6^ cells/the volume of added virus suspension, expressed as infectious units per millilitre (IU/ml).

### Transduction of gene-modified T cells

CD8^+^ T cells were seeded at a concentration of 1×10^6^ cells/ml in 6-well culture plates in the presence of 100 u/ml IL-2 and 50 ng/ml OKT3 (Ortho Biotech, Raritan, NJ, USA) 48 hrs prior to transduction. The fresh recombinant viral concentrate containing 6 μg/ml polybrene (Sigma-Aldrich) was added and incubated at 37°C, 5% CO_2_ for 4 hrs before the culture supernatant was replaced by fresh medium supplemented with IL-2 and OKT3 to dilute the polybrene to 2 μg/ml. A second transduction was performed 2 days later. Five days later, gene-modified CD8^+^ T cells were collected to detect the expression of GFP and functional activities.

### Immunofluorescence

The expression of GFP of engineered CD8^+^ T cells were examined by fluorescence microscope (Nikon Corporation, Tokyo, Japan) and flow cytometry. The untranduced CD8^+^ T cells were served as negative control.

### Cytokine production

Dendritic cells (DCs) were induced from the homologous PBMCs and cultured as previously described 37. DCs loaded or unloaded with peptides were co-cultured with effector T cells in 96-well plates (Nunc). To determine the activity of the TCR gene-modified T cells upon antigen stimulation, ten groups were set in the experiments: (1) MTB/HIV Td + DC group: TETARs+ DCs unloaded with peptide; (2) UnTd + DC-Ag85B_199–207_ group: untransduced T cells + Ag85B_199–207_ -loaded DCs; (3) UnTd + DC-Env_120–128_ group: untransduced T cells + Env_120–128_ -loaded DCs; (4) EmTd + DC-Ag85B_199–207_ group: empty vector-transduced T cells + Ag85B_199–207_ -loaded DCs; (5) EmTd + DC-Env_120–128_ group: empty vector-transduced T cells + Env_120–128_-loaded DCs; (6) MTB/HIV Td + DC-PP65_495–503_ group: TETARs+ HLA-A*0201-restricted CMV PP65_495–503_ (NLVPMVATV)-loaded DCs; (7) MTB Td + DC-Ag85B_199–207_ group: MTB/TCR transduced T cells + Ag85B_199–207_ -loaded DCs; (8) MTB/HIV Td + DC-Ag85B_199–207_ group: TETARs + Ag85B_199–207_-loaded DCs; (9) HIV Td + DC-Env_120–128_ group: HIV/TCR transduced T cells + Env_120–128_-loaded DCs; (10) MTB/HIV Td + DC-Env_120–128_ group: TETARs + Env_120–128_-loaded DCs. In some assays, DCs were transfected with the pCAGGS-Env plasmid (gifted by Dr. James M. Binley in Torrey Pines Institute for Molecular Studies, San Diego, CA, USA) or loaded with ovalbumin (OVA) antigen (Sigma-Aldrich). The E/T = 7 in IFN-γ assays and = 20 in TNF-α assays respectively. The culture supernatants were harvested 18 hrs later for detecting secretion of IFN-γ and 24 hrs later for TNF-α using ELISA kits (Bender MedSystems, Vienna, Austria) as per the manufacturer's instructions. Both Effector/Target ratios and incubation time were determined according to our previous study 26.

### Cytotoxicity assays

Cytolytic activity of transduced T cells was measured by a DELFIA EuTDA cytotoxicity kit (Perkin-Elmer Life Sciences, Norwalk, CT, USA) as described previously 26. Groups were set up as described above. DCs loaded with Ag85B_199–207_, Env_120–128_, PP65_495–503_ or unloaded DCs were served as target cells, and were co-cultured with engineered CD8^+^ T cells at the E:T ratio of 30:1. Four hours later, supernatants were collected to detect the cytolytic activity. The released fluorescence by lytic cells was read using Wallac Victor 2 Multilabel Counter (Perkin-Elmer). Per cent-specific lysis was calculated as follows: 100 × [(experimental release − spontaneous release)/(maximum release − spontaneous release)], where the spontaneous release was determined by reading the values of the target cells alone, and the maximum release was determined by completely lysing labelled target cells.

### Statistics

Differences in cytokine production and cytotoxicity among the 10 groups were analysed by one-way analysis of variance (anova) and multiple comparison tests (LSD or Dunnett's T3). *P* values were two-sided, and *P* values <0.05 were considered statistically significant. All statistical analyses were performed using the SPSS version 17.0 for windows statistical package (SPSS, Chicago, IL, USA).

## Results

### Screening for MTB or HIV-1 peptide-specific TCRs

**Fig. 1:**
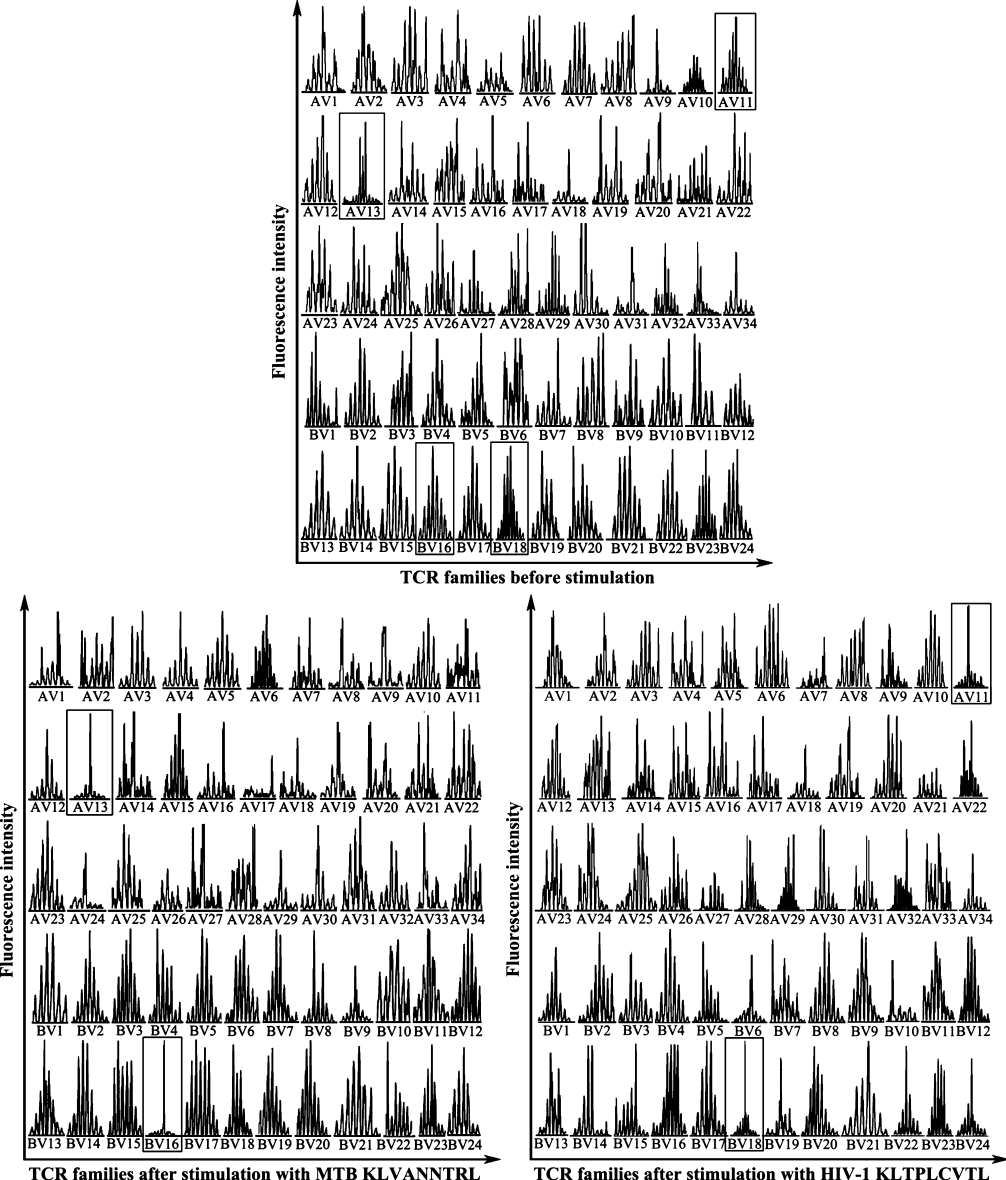
CDR3 spectratypes of 34 TCR Vα and 24 Vβ gene families from CD8^+^ T cells before and after antigen peptide stimulation. The spectratype of the MTB Ag85B_199–207_-stimulated CD8^+^ T cell TCR Vα13/Vβ16 gene families and the HIV-1 Env_120–128_-stimulated CD8^+^ T cell TCR Vα11/Vβ18 gene families (framed) show a single peak after stimulation indicating specificities for corresponding peptides. AV, Vα; BV, Vβ.

CDR3 spectratypes of all TCR Vα and Vβ gene families exhibited a Gaussian distribution of eight peaks or more prior to stimulation, demonstrating polyclonal proliferation and polyfamilies of the normal TCR repertoire. However, a few Vα and Vβ gene family CDR3 spectratypes showed skewed oligo-peaks, or even single-peak distributions after stimulation. Unimodal distribution results from monoclonal expansion of T cells following antigen stimulation indicating that the corresponding gene families were peptide-specific. Consequently, the TCR Vα13 and Vβ16 gene families expressed by CD8^+^ T cells were confirmed to be MTB Ag85B_199–207_-specific, and the Vα11 and Vβ18 to be HIV-1 Env_120–128_-specific (Fig. 1).

### Construction of recombinant retroviral vectors

**Fig. 2:**
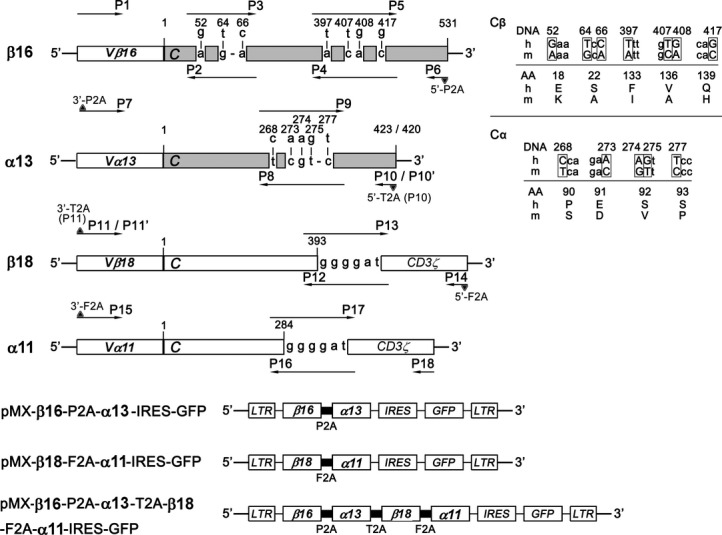
Construction of a retroviral vector expressing MTB Ag85B_199–207_-specific TCR and/or HIV-1 Env_120–128_-specific TCR genes. Nine critical amino acids in the constant (C) regions of β16 and α13 (specific to Ag85B_199–207_) were replaced by their murine counterparts. The CD3ζ gene was ligated to each downstream β18 and α11 gene sequence (specific to Env_120–128_) to substitute partial C regions. Gene fragments were linked by different 2A peptides and cloned into the pMX-IRES-GFP retroviral vector. Cβ, β chain C region; Cα, α chain C region; DNA, the DNA sequence; AA, the amino acid sequence; h, human; m, murine; IRES, internal ribosomal entry site; GFP, green fluorescent protein.

To achieve optimal expression of the two transferred TCRs and to minimize mispairing among exogenous and endogenous TCR chains, we replaced nine critical amino acids in the C regions of the α and β chains of MTB/TCR by their murine counterparts, and substituted original C domains downstream of the extracellular cysteine of the HIV/TCR α and β chains with a complete human CD3ζ sequence. The above four gene segments were linked by three different 2A peptides as a single transcript then cloned into a retroviral vector to obtain the recombinant vector pMX-β16-P2A-α13-T2A-β18-F2A-α11-IRES-GFP with the equal and stable expression of the inserted gene segments. Similarly, recombinant vectors carrying MTB/TCR (pMX-β16-P2A-α13-IRES-GFP) and HIV/TCR (pMX-β18-F2A-α11-IRES-GFP) genes were also constructed (Fig. 2). The infectious viral titres of the above three viral vectors were 8 × 10^6^, 1.59 × 10^7^, 2.14 × 10^7^ IU/ml respectively (data not shown).

### Expression of GFP by TCR gene-modified T cells

**Fig. 3:**
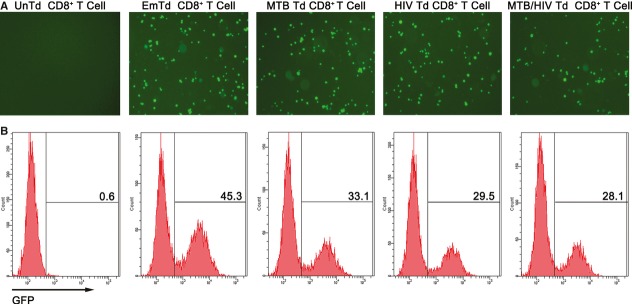
GFP expression of TCR gene-modified CD8^+^ T cells. Expression was observed under fluorescence microscope (**A**) and analysed by FACS (**B**). UnTd, untransfected; EmTd, transfected with empty retrovirus; MTB Td, transfected with retrovirus carrying MTB Ag85B_199–207_-specific TCR genes; HIV Td, transfected with retrovirus carrying HIV-1 Env_120–128_-specific TCR genes; MTB/HIV Td, transfected with retrovirus carrying both TCR genes.

Green fluorescence was clearly detected in TCR gene-modified T cells but not in untransduced T cells 5 days after transfection. GFP expression was measured by flow cytometry demonstrating that 28–34% of the gene-modified T cells expressed GFP compared to 0.6% GFP expression in untransduced T cells (Fig. 3).

### Secretion of IFN-γ and TNF-α by TETARs in a peptide-specific manner

**Fig. 4:**
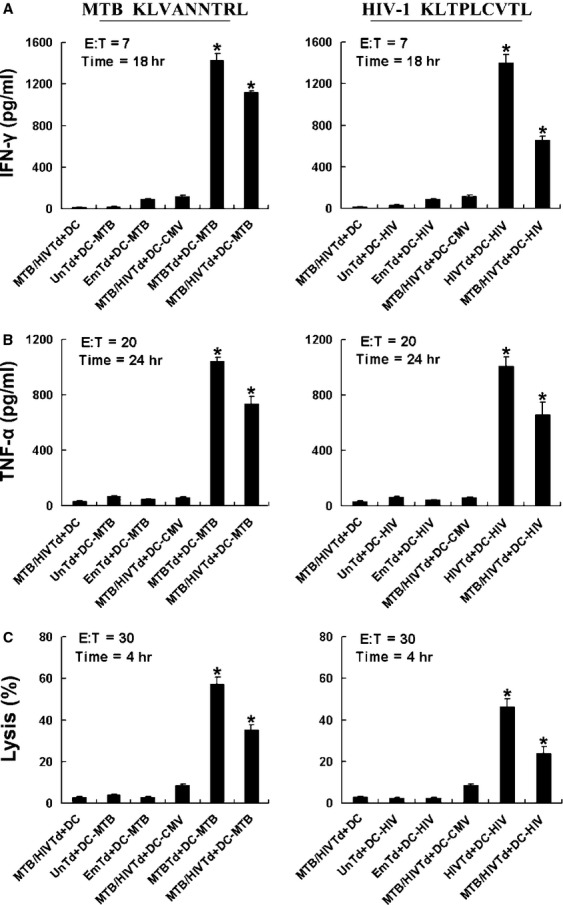
Cytokine secretion and cytotoxicity of TCR gene-modified CD8^+^ T cells. Left panels, CD8^+^ T cells co-cultured with DCs loaded with MTB Ag85B_199–207_ (KLVANNTRL); right panels, CD8^+^ T cells co-cultured with DCs loaded with HIV-1 Env_120–128_ (KLTPLCVTL). Data represent average values of three independent experiments ± SE. *P < 0.05 compared to UnTd + DC-Ag85B_199–207_ group (left panel) or UnTd + DC-Env_120–128_ group (right panel). DC-MTB: DCs loaded with MTB Ag85B_199–207_; DC-HIV: DCs loaded with HIV-1 Env_120–128_; DC-CMV: DCs loaded with CMV PP65_495–503_ (NLVPMVATV).

When co-cultured with DCs loaded with MTB Ag85B_199–207_, the levels of IFN-γ and TNF-α produced by MTB/TCR gene-modified T cells and TETARs were significantly higher than untransduced or empty vector-transduced T cells (*P* < 0.05), indicating that these T cells possessed enhanced activity after TCR gene modification. Meanwhile, TETARs co-cultured with DCs loaded with PP65_495–503_ did not show any increased activity compared to co-cultures with unloaded DCs (*P* > 0.05), suggesting that the enhanced activity following TCR gene modification was an antigen peptide-specific one(Fig. 4A and B).

Similarly, HIV/TCR gene-modified T cells and TETARs exerted significantly elevated cytokine secretion levels following HIV-1 Env_120–128_ peptide stimulation compared to untransduced or empty vector-transduced T cells (*P* < 0.05). This activity was also antigen peptide-specific because the PP65_495–503_ peptide could not increase cytokine production by TETARs compared to cytokine production observed for unstimulated cells (*P* > 0.05) (Fig. 4C).

### Peptide-specific cytolytic activity of TETARs

Besides the capacity to specifically release IFN-γ and TNF-α, TETARs also possessed increased cytolytic activity. Per cent-specific lysis of DCs loaded with MTB Ag85B_199–207_ peptide or HIV-1 Env_120–128_ peptide by TETARs was significantly higher (35.162 ± 2.670% and 23.885 ± 3.257% respectively) than the ratio observed when the effector cells were untransduced or were empty vector-transduced (*P* < 0.05). This lysis ratio was even higher following co-culture with MTB/TCR gene-modified T cells or HIV/TCR gene-modified T cells. However, there was no significant difference in the per cent-specific lysis mediated by TETARs of DCs loaded with PP65_495–503_ peptide and of unloaded DCs (*P* > 0.05) (Fig. 4).

### Activities of TETRAs against endogenous antigens

**Fig. 5:**
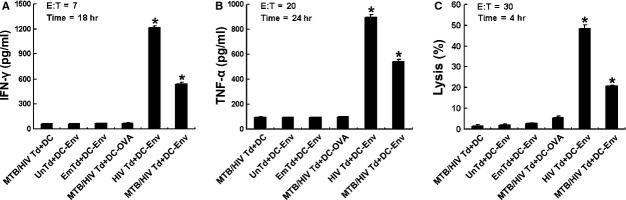
Immune responses to endogenous antigens by TCR gene-modified CD8^+^ T cells. Activities of T cells were analysed by ELISA of IFN-γ (**A**) and TNF-α (**B**) as well as DELFIA assay (**C**). DC-Env, DC-OVA: DCs transfected with the Env- or OVA-expressing plasmid.

To further determine the activity of TCR gene-modified T cells against the intracellular infectious pathogens, DCs transfected with the Env-expressing plasmid were used as the target cells. As expected, both HIV/TCR gene-modified T cells and TETARs showed significantly enhanced activities when exposed to the endogenously presented antigens by DCs. The significantly higher levels of cytokine secretion as well as cytolytic activity by TETRAs were observed when compared with T cells without TCR gene modification or without specific antigen presentation (*P* < 0.01). Similar to the peptide stimulation described above, HIV/TCR gene-modified T cells showed even higher activities against DCs presenting endogenous Env antigen (*P* < 0.01) (Fig. 5).

## Discussion

T cell-mediated immune responses are essential to the control of infections caused by intracellular pathogens, including MTB and HIV. HIV infection, which substantially reduce the CD4^+^ T cell numbers (and function) in peripheral tissues resulting in loss of granuloma integrity and MTB containment thereby facilitating MTB/HIV co-infections 38, 39. In MTB/HIV co-infected individuals, development of MTB-specific T helper type 1 responses, the activity of antigen-specific CTLs, and cytokine production were significantly impaired 4, 40 representing major factors responsible for the failure of the immune system to inhibit pathogen replication and dissemination. CD8^+^ CTL immune response plays a critical role in controlling MTB and HIV-1 replication; therefore, augmenting CTL responses should facilitate inhibiting MTB and HIV-1 replication thereby improving the clinical course of disease.

Using adoptive transfer of *ex vivo*-expanded autologous CD8^+^ CTLs, Lieberman *et al*. demonstrated that CD4^+^ T cell counts increased, plasma viraemia decreased and HIV-specific CTL activity increased in HIV patients; however, this treatment had minimal effects on decreasing viral loads and other surrogate markers of viral replication 41. The minimal effect of the adoptively transferred autologous CTLs on disease course was possibly as a result of the compromised function and decreased replicative capacity of CTLs derived from HIV-1-infected individuals 42.

An effective technology used for generating HIV-1-specific CTLs capable of delivering effective immunity involves genetic transfer of HIV-1-specific TCR α and β chain genes into peripheral autologous CD8^+^ T lymphocytes; an approach which has recently been demonstrated to generate potent *in vitro* and *in vivo* HIV-1-specific activity 27. To overcome immune escape of rapidly mutating HIV-1 isolates, adoptive TCR transfer was used by Hofmann *et al*. who transduced CD8^+^ T cells with TCRs specific for two HIV-1 epitopes. The resulting dual-TCR gene-modified CD8^+^ T cells responded to stimulation by either antigen resulting in cytokine secretion and cytotoxic activity 28, suggesting that TCR gene therapy could be adapted for use as an HIV immunotherapy.

Adoptive transfer of inactivated MTB-sensitized autologous T cells has previously been demonstrated to contribute to MTB clearance in multi-drug-resistant TB patients 43, 44. However, few effector T cells exist in TB patients and these cells are difficult to amplify to levels sufficient for adoptive transfer thereby limiting the clinical use of this approach. By using TCR gene modifications, significantly increased number of antigen-specific effector T cells can be generated in a short time. We previously demonstrated that TCR genetically engineered CD4^+^ and CD8^+^ T cells recognized MTB antigens specifically and displayed superior effector functions 26, suggesting that TCR gene therapy might be a potential TB immunotherapy approach.

To promote immune protection against MTB and HIV in co-infected patients, adoptive transfer of TCR gene-modified T cells represents a promising strategy not yet described in the context of MTB/HIV co-infections even though this approach increases lymphocyte numbers and proliferative responses as well as functional avidity. As both MTB and HIV-1 are susceptible to high mutation rates, transduction of TCRs specific for conserved MTB and HIV-1 epitopes as a means of generating T cells with dual pathogen specificities should elicit immune responses with the potential of efficiently clearing infections by either or both pathogens.

Ag85B is a member of the secreted extracellular Ag 85 protein family which occupies nearly 30% of MTB culture filtrate proteins 45. Members of Ag 85 are all associated with mycolyltransferase activity *in vitro* 46 and play important roles in early protective immunity induction 47, 48. The Ag85B_199–207_ peptide is a naturally processed and highly conserved MTB epitope widely recognized by humans possessing the HLA-A*0201 allele and is associated with the induction of CTL response against MTB accompanied by production of proinflammatory cytokines IFN-γ and TNF-α. Immunization of HLA-A2/K^b^ transgenic mice with Ag85B_199–207_ elicited increased levels of T cell proliferation and cytotoxicity. Furthermore, Ag85B_199–207_-specific CD8^+^ T cells were able to lyse HLA-A*0201^+^ peptide-pulsed autologous DCs and respond to BCG-infected macrophages, demonstrating the presence of CD8^+^ HLA-A*0201-restricted T cells in the human T cell repertoire and the high efficiency of recognizing MTB-infected macrophages 49. Development of HIV-specific CTL responses to many viral proteins including the gp160 envelope protein 50, 51 appears to coincide with the early suppression of virus replication 52. The intracellularly processed HIV-1 Env_120–128_ peptide (KLTPLCVTL) maps to the N-terminal regions of gp160 53 and is highly conserved among HIV-1 B subtype strains, appearing in 80% of variant HIV-1 epitope sequences and induces human HLA-A*0201^+^ individuals to secrete high levels of IFN-γ 52, 54. In the class I-restricted cellular immune responses to heterogenous Env-derived epitopes, Env_120–128_ is one of the two peptides that CTL activities of HIV-infected HLA-A2^+^ patients are mainly directed against 52. By transferring CD8^+^ T cells with the Ag85B_199–207_- and the Env_120–128_-specific TCR genes simultaneously we obtained CTLs capable of recognizing both MTB and HIV-1 epitopes.

To prevent TCR gene mismatches several strategies were used in the generation of TETARs, including introduction of C region point mutations, substitution of partial C regions with the CD3ζ gene and usage of three different 2A peptides to separate the four TCR chains. The effects of the gene transduction experiments were characterized using functional T cell assays. Promoting correct pairing of HIV/TCR α and β chains by substituting partial C regions with the CD3ζ gene further ensured that all TETAR TCRs transmitted similar activation signals. This is because of the fact that TCRαβ:CD3ζ will not competitively bind to endogenous CD3 molecules thereby preventing compromise of expression and function of existing TCRs 30. For these reasons, involvement of the CD3ζ gene as part of the TCR gene transfer strategy is important to the generation of functional TCR gene-modified T cells, especially those expressing more than two distinct TCRs.

Comparison of the anti-MTB and anti-HIV-1 avidity among TETARs and single TCR gene-transferred T cells demonstrated that TETARs had lower levels of cytokine production and cytotoxicity than that single TCR gene-transferred T cells. The underlying reasons may include the following: (1) exogenous TCRs and endogenous TCRs competed for limited space on the cell surface resulting in reduced expression of transferred TCRs on the TETAR surface, or (2) the TCRαβ:CD3ζ occupied a larger synaptic size further reducing the area of cell surface expression 55. A sufficient number of TCRs are needed for T cell activation 56, and lower expression levels of each TCR on dually gene-transferred T cells made TETARs more difficult to be sensitized than single TCR gene-transferred T cells. However, TETARs still exerted significantly increased peptide- and antigen- specific cytokine secretion and cytolytic activities compared to un-transduced or empty vector-transduced T cells regardless of pathogen specificity. Previous studies have demonstrated that simultaneous introduction of multiple TCR genes affected transduction efficiency 57, and equal expression of the transgenes when compared to expression of genes following multiple transductions. Improving TETARs efficacy by silencing endogenous TCR expression will be the subject of future work 22, 58.

In conclusion, we generated human TETARs by simultaneously introducing genes encoding TCRs specific for MTB and HIV using a 2A ribosomal skip element with a retroviral vector. The TETARs generated as a result of this approach retained specificity to peptides from different pathogens and could be stimulated by either peptide or antigen to produce cytokines and elicit cytotoxic responses. To the best of our knowledge, this is the first report describing the generation of T cells expressing TCRs with specificity for epitopes expressed by two distinct pathogens. Dual specificities will ensure recognition of pathogen epitopes by adoptively transferred TETARs despite antigen mutations observed in one pathogen. Our research provides new insights into TCR gene therapies developed by introducing multi-pathogen, epitope-specific TCR gene-modified T cells that can be used as an adoptive immunotherapy approach for treating MTB/HIV co-infected patients.
